# Microsatellite DNA Analysis of Genetic Diversity and Parentage Testing in the Popular Dog Breeds in Poland

**DOI:** 10.3390/genes12040485

**Published:** 2021-03-26

**Authors:** Anna Radko, Angelika Podbielska

**Affiliations:** Department of Animal Molecular Biology, National Research Institute of Animal Production, Krakowska 1, 32-083 Balice, Poland; angelika.podbielska@izoo.krakow.pl

**Keywords:** STR, domestic dog, biodiversity, individual identification, parentage

## Abstract

There is growing concern that extreme breed standardization contributes to a reduction of the effective population size and high levels of inbreeding, resulting in the loss of genetic diversity in many breeds. This study examined genetic diversity among eight popular dog breeds in Poland and evaluated the effectiveness of a 21-microsatellite (STR) panel recommended by the International Society for Animal Genetics (ISAG) for parent verification. The following breeds were characterized: German Shepherd, Maltese, Irish Wolfhound, Yorkshire Terrier, Biewer Yorkshire Terrier, Golden Retriever, Labrador Retriever, and French Bulldog. STRUCTURE analysis showed breed distinctiveness among all the dog breeds under study. Reynold’s distance ranged between θ_w_ = 0.634 and θ_w_ = 0.260. The studied breeds showed a medium level of genetic differentiation; the mean number of alleles per locus ranged from 3.4 to 6.6, and the effective number of alleles from 2.1 to 3.5. The mean degree of heterozygosity varied from 49% to 69% and from 47% to 68% for H_O_ and H_E_, respectively. The population inbreeding coefficient (FIS) indicated an absence of inbreeding in the studied breeds. The average polymorphism information content (PIC) values for most of the breeds were higher than 0.5. The cumulative power of discrimination (PD) for all the markers in all breeds reached high values (close to 1.0), while the probability of identity (P_ID_) was low, ranging between 10^−11^ and 10^−19^. The cumulative exclusion probability when the genotypes of one (PE_1_) and both parents (PE_2_) are known and showed that the parentage can be confirmed with a probability of 94.92% to 99.95% and 99.78% to 99.9999%, respectively.

## 1. Introduction

The dog (*Canis familiaris*) is one of the first animals domesticated by man and has accompanied humans in their day-to-day life for thousands of years. Historically, dogs were used as working animals to herd livestock, hunt, and guard the home, and now they are also treated as companion animals [1,2,3]. Their extensive use is associated with the human selection of dogs for certain phenotypes. Strong selection for desirable traits often causes an irreversible loss of alleles and a reduction of genetic diversity, which may produce undesirable characteristics and health problems [3,4,5]. Around the world, more than 400 breeds of dogs are recognized [1]. The American Kennel Club (AKC) currently registers 197 dog breeds, and the Fédération Cynologique Internationale (FCI) officially recognizes 353 breeds officially [6,7]. Breeding to achieve specific breed standards can lead to a reduction in effective population size and result in increased levels of inbreeding within breeds, resulting in a loss of genetic diversity in many breeds [5,8]. Therefore, it is important to carry out selective breeding while maintaining breed purity and high biodiversity. To this end, the genetic structure of different dog breeds needs to be studied and genetic changes occurring in breeds have to be monitored.

Microsatellite (STR) markers are a well-known effective and powerful tool widely used to investigate the genetic structure and diversity of dog breeds [9,10,11,12,13]. They are also the most important markers used for dog identification and parentage verification [9,14,15]. A panel of 21 markers recommended for parentage testing in domestic dogs by the International Society for Animal Genetics (ISAG) [16] is the panel standardized across multiple laboratories for canine genotyping and used for routine pedigree testing [14]. The analysis of polymorphism was carried out in the following breeds of dogs in Poland: Polish Hunting Dog [13] and Polish Greyhound [17] based on 21 STR recommended by ISAG, and in Borzoi and Tatra Shepherd Dog based on 18 STR [9,17]. No information is available regarding genetic variation among the most popular breeds of dogs in Poland, namely, German Shepherd, Yorkshire Terrier, Golden Retriever, Labrador Retriever, and French Bulldog [18]. In our study, the genetic analysis of these breeds was possible based on data collected as part of pedigree testing conducted at the National Research Institute of Animal Production (NRIAP). Additional analyses included Irish Wolfhound, Biewer Yorkshire Terrier, and Maltese, which are tested in fairly large numbers as part of parentage control. The objective of the study was to examine genetic variation within and among these dog breeds and to evaluate the effectiveness of an STR panel recommended for parentage testing.

## 2. Materials and Methods

The study was conducted based on the results of analysis of microsatellite polymorphism performed as part of canine pedigree testing at the National Research Institute of Animal Production in 2018–2020. A total of 903 samples were used from eight breeds, namely, German Shepherd (GS, *n* = 260), Maltese (M, *n* = 81), Irish Wolfhound (IW, *n* = 86), Biewer Yorkshire Terrier (BYT, *n* = 131), Yorkshire Terrier (YT, *n* = 77), Golden Retriever (GR, *n* = 48), Labrador Retriever (LR, *n* = 103), and French Bulldog (FB, *n* = 117). For all breeds, sampling was a selection of at least 70 unrelated animals (males and females) from different kennels, except for the GR breed for which 48 samples were collected.

This analysis uses the core panel of STR markers recommended by ISAG for individual identification and parentage analysis and a gender identification marker. The following microsatellite markers were used: AHTk211, CXX279, REN169O18, INU055, REN54P11, INRA21, AHT137, REN169D01, AHTh260, AHTk253, INU005, INU030, FH2848, AHT121, FH2054, REN162C04, AHTh171, REN247M23, AHTH130, REN105L03, REN64E19, and Amel locus. DNA was extracted from swabs and blood using the Sherlock AX Kit (A&A Biotechnology, Gdynia, Poland), following the manufacturer’s protocol. The extracts were quantified with a NanoDrop 2000 spectrophotometer (Thermo Scientific, Wilmington, DE, USA). The STR loci were amplified using Phusion U Hot Start DNA Polymerase (Thermo Scientific, Wilmington, DE, USA), and the PCR reaction was performed on Veriti^®^ Thermal Cycler amplifier (Applied Biosystems, Foster City, CA, USA), using the following thermal profile: 5 min of initial DNA denaturation at 98 °C, followed by 30 cycles of denaturation at 98 °C for 15 s, annealing at 58 °C for 75 s, elongation of starters at 72 °C for 30 s, and final elongation of starters at 72 °C for 5 min. Analysis of the obtained PCR products was performed using an ABI 3130*xl* capillary sequencer (Applied Biosystems, Foster City, CA, USA). The amplified DNA fragments were subjected to electrophoresis in 7% denaturing POP-7 polyacrylamide gel in the presence of a standard length of 500 Liz and a reference sample. The results of the electrophoretic separation were analyzed automatically using the GeneMapper^®^ Software 4.0 (Applied Biosystems, Foster City, CA, USA).

### Data Analysis

Population structure was analyzed using STRUCTURE software version 2.3.3 [19] considering an admixture model with correlated allele frequencies between breeds. The length of the burn in and Markov chain Monte Carlo (MCMC) simulations was 50,000 and 100,000, respectively, in 10 runs for each number of clusters (K) ranging between 4 and 10. The results were exported to STRUCTURE HARVESTER [20] for plotting the likelihood membership coefficient (DeltaK) values so as to determine the most likely number of clusters. Genetic distance was analyzed using Reynolds’s distance—θ_w_ [21].

(1) $$\theta_{w}=\sqrt{\frac{\sum_{l}\sum_{u}{\left(X_{u}-Y_{u}\right)}^{2}}{2\sum_{l}\left(1-\sum_{u}X_{u}-Y_{u}\right)}},$$

where X_u_, Y_u_ are allele frequencies from the first and second populations.

Based on this genetic distance, an unweighted pair-group method with averages (UPGMA) dendrograms were constructed to illustrate the similarities between the populations. Observed heterozygosity—H_O_, expected heterozygosity—H_E_, and inbreeding coefficient—F_IS_, were calculated according to Nei and Roychoudhury [22], and Wright [23]. The Hardy–Weinberg equilibrium (HWE) of the 21-STR loci was tested by exact test using an algorithm based on Markov Chain Monte Carlo methods [24]. The genetics parameters were calculated as follows:

1. Polymorphic information content—PIC [25],
2. Power of discrimination—PD [26],
  (2) $$P_{D}=1-\overset{n}{\underset{j=k}{\sum }}\overset{n}{\underset{k=1}{\sum }}p^{2}\mathrm{jk}$$
  (3) $$\mathrm{CPD}=1-\overset{n}{\underset{i=1}{\prod }}\left(1-P_{\mathrm{Di}}\right),$$
  where p_jk_ is the allele frequency j,k for i-locus; CPD is the cumulative power of discrimination.
3. Probability of identity—P_ID_ [27],
  (4) $$P_{\mathrm{ID}}=\sum p_{i}^{4}+\sum \sum {\left(2p_{i}p_{j}\right)}^{2}$$
  (5) $${\mathrm{CP}}_{\mathrm{ID}}=\prod P_{\mathrm{ID}}\;,$$
  where p_i_ p_,j_ is allele frequencies j,i; CP_ID_ is the cumulative probability of identity.
4. Probability of parentage exclusion for each locus, when the genotypes of one and both parents are known (PE_1_ and PE_2_) and the cumulative probability of parentage exclusion (CPE) [28],
  (6) $${\mathrm{PE}}_{1}=\overset{n}{\underset{i=1}{\sum }}p_{i}^{2}{\left(1-p_{i}\right)}^{2}+\overset{n}{\underset{i>j=1}{\sum }}2p_{i}p_{j}{\left(1-p_{i}+p_{j}\right)}^{2}$$
  (7) $${\mathrm{CPE}}_{1}=1-\overset{n}{\underset{i=1}{\prod }}\left(1-{\mathrm{PE}}_{1i}\right)$$
  (8) $${\mathrm{PE}}_{2}=\overset{n}{\underset{i=1}{\sum }}p_{i}{\left(1-p\right)}^{2}-\overset{n}{\underset{i>j=1}{\sum }}{\left(p_{i}p_{j}\right)}^{2}\left[4-3\left(p_{i}+p_{j}\right)\right]$$
  (9) $${\mathrm{CPE}}_{2}=1-\overset{n}{\underset{i=1}{\prod }}\left(1-{\mathrm{PE}}_{2i}\right)\;,$$
  where p_jk_ is allele frequency j,k for i-locus; CPE_1_, CPE_2_ are cumulative probabilities of identity.

The statistical analysis was performed using the IMGSTAT software, ver. 2.10.1 (2009), which supports the laboratory of the National Research Institute of Animal Production.

## 3. Results

A total of 185 alleles were detected at 21 STR loci across all breeds. The total number of alleles per locus ranged between 6 for AHTk211 and 12 for AHTk137 and REN169018.

### 3.1. Breed Relationships

**Figure 1:**
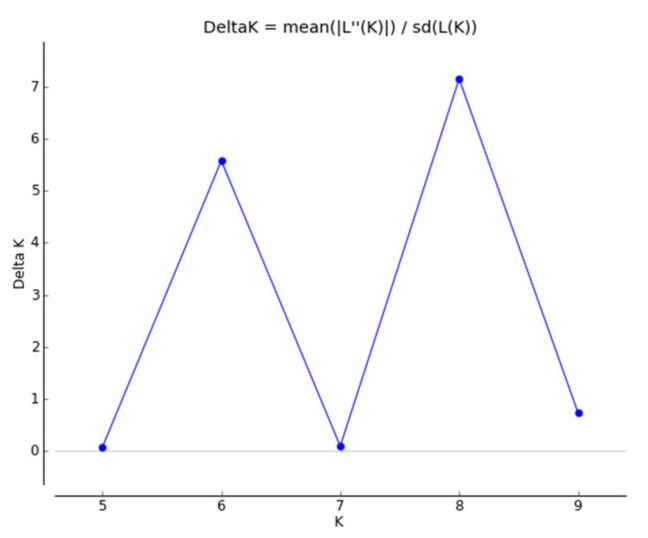
Delta K values for STRUCTURE analysis of dog breeds obtained by the program Structure Harvester.

The genetic population structure of each breed was determined based on the admixture level for each individual dog using the correlated allele frequencies model implemented within the STRUCTURE software. The results of Delta K indicated that the optimal number of genetic clusters representing most similar individuals for breeds was at K = 8 (Figure 1). The average proportion of assignment to the cluster above 90% was found for five breeds, i.e., GS (98%), GL (97%), IW and FB (96%), and M (93%). A lower assignment value was found in the BYT breed (88%) and the lowest in the LR and YT (85%).

**Figure 2:**
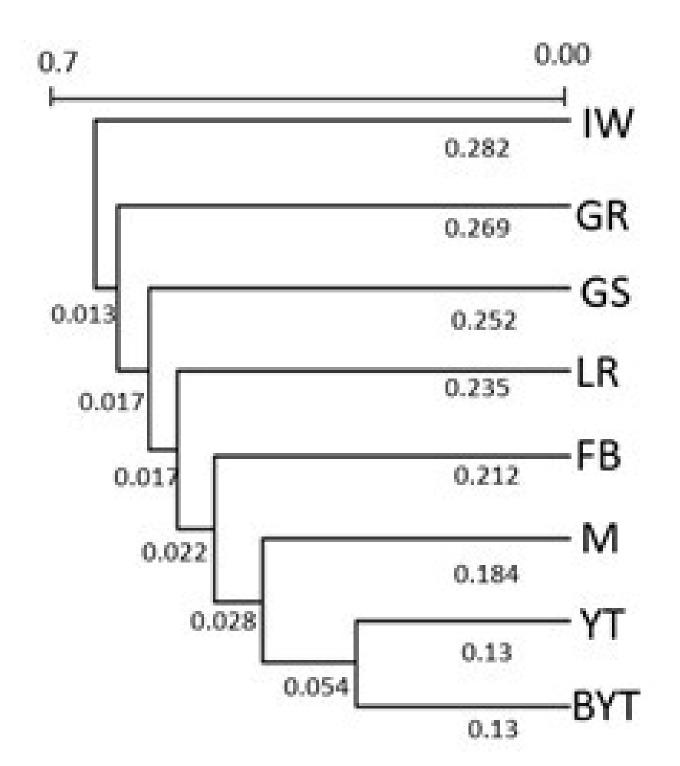
Dendrogram of Reynolds genetic distance between dog breeds by unweighted pair-group method with averages (UPGMA) algorithm.

Reynold’s (1983) genetic distance between eight breeds is summarized in Table 1. The genetic distance was the greatest between GS and IW (θ_w_ = 0.634), and the smallest between BYT and YT (θ_w_ = 0.260). The UPGMA dendrogram revealed that the YT and BYT were grouped together, and the Irish Wolfhound breed was farthest away from others (Figure 2).

### 3.2. Diversity Analysis

The greatest number of alleles was noted for the breeds BYT (138 alleles) and M (135 alleles), and the smallest for IW (72 alleles). The mean number of alleles per locus for breeds ranged from 3.4 for IW to 6.6 for BYT, whereas the effective number from 2.1 for WI to 3.5 for M and YT (Table 2).

Out of the 185 alleles found within these breeds, 20 were breed specific (Table 3). The greatest number of private alleles was observed at the locus REN169O18 (four alleles). There were two alleles each at REN247M23 and REN105L03, and one allele each at 12 loci. No breed-specific alleles occurred at the remaining six loci—AHTk211, INU055, REN169D01, INU030, AHTh171, FH2054. The greatest number of private alleles was identified in the GS breed. Specific alleles for this breed occurred at six loci, yet in most cases with low frequencies (<0.09), only two alleles occurred with frequencies of 0.198 and 0.217 for 212 bp allele in REN162C04 and 158 bp in REN169018, respectively. In FB, a higher frequency of 0.12 and 0.218 was noted for the 229 bp allele in REN105L03, and the 156 bp allele in REN169O18, respectively. In the YT and BYT breeds in CXX279, there was a 128 bp allele with a frequency exceeding 16% each, which did not occur in any of the other breeds. The other private alleles exhibited low frequency (less than 3%). No breed-specific alleles were found in the Irish wolfhound. The frequencies of the breed-specific alleles are presented in Table 3.

Estimates of within-breed genetic diversity are summarized in Table 4. The highest average heterozygosity was found for M (Ho = 0.685 and H_E_ = 0.677), YT (Ho = 0.662 and H_E_ = 0.698), and BYT (Ho = 0.661 and H_E_ = 0.658). Among the eight dog breeds considered, the lowest values of heterozygosity were found for IW (Ho = 0.491 and H_E_ = 0.474). The population inbreeding coefficient (F_IS_) ranged from −0.049 (GR) to 0.053 (YT). The average *p*-value of HWE for each breed was higher than 0.05 (Table 4).

### 3.3. Parentage Testing and Individual Identification

**Figure 3:**
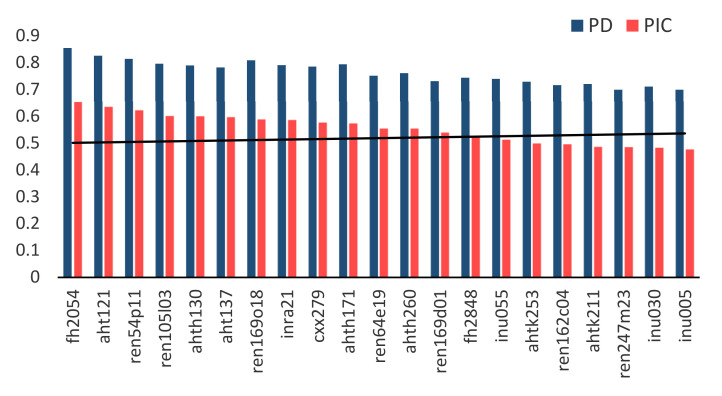
Mean values of the polymorphic information content (PIC) and power of discrimination (PD) for the eight dog breeds under study.

The parameters for determining the suitability of the analyzed STR for identification and parentage testing are presented in Table 4. Polymorphism exceeding 0.6 was observed only for five STR (AHT121, FH2054, AHTh171, REN54P11, and REN64E19), while a level over 0.5 was detected for 10 markers. The lowest polymorphism below 0.4 was noted for the rest of the loci. Mean PIC values for the studied breeds varied between 0.414 (for IW) and 0.655 (YT). The mean PD values for individual markers, calculated for all the eight dog breeds together, exceeded 0.8 for AHT121, FH2054, REN54P11, and REN169O18. For the other loci, PD had mean values exceeding 0.7 or close to 0.7 (REN247M23 and INU005) (Figure 3). The power of discrimination for the whole set of 21 STR, for each of the breeds, shows the high values of 0.999999999874911 (IW) to close to 1.0 (BYT, TY).

The cumulative probability of identity for 21 STR loci resulted in values as low as 4.5 × 10^−19^, 2.3 × 10^−19^ and 6.8 × 10^−19^ for M, YT, and BYT breeds, respectively. The higher values of 1.8 × 10^−13^ and 6.7 × 10^−11^ were obtained for GS and IW breeds, respectively (Table 4). The panel of 21 microsatellite markers was assessed for their power of exclusion to test parentage in dogs of eight breeds. The probabilities of exclusion were calculated for two hypothetical situations with one parental genotype available (PE_1_) and two parental genotypes available (PE_2_). The probability of exclusion for one parent available (PE_1_) ranged between 0.005 (REN169DO1 in IW) and 0.467 (CXX279 in YT) and when two parents were available (PE_2_) between 0.047 (REN169DO1 in IW) and 0.642 (REN169DO1 in YT) across different markers and breeds. The cumulative exclusion probability for PE_1_ and PE_2_ varied from 0.949242 (IW) to 0.999495 (YT) and from 0.997768 (IW) to 0.999999 (YT), respectively (Table 4).

## 4. Discussion

As a consequence of selection pressure, management in closed populations, and historical bottlenecks, many dog breeds are exposed to an increase in inbreeding and a loss of diversity [5,29]. It is therefore essential to analyze the genetic structure and evaluate the genetic variation of as many dog breeds as possible and keep track of changes in these breeds. It is also necessary to evaluate the usefulness of DNA markers used for identification and parentage testing of dogs.

With the advancement of science and technology, new genetic markers, such as single nucleotide polymorphisms, have become widely used; however, due to the cost and time of analysis, STR typing is still used in biodiversity and kinship analysis [8,9,10,11,12,13,14,15,17].

**Figure 4:**
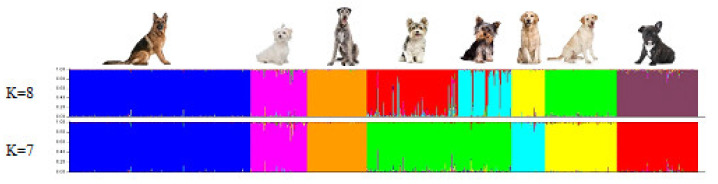
STRUCTURE analysis of 21 STR genotypes from all study dogs (903 samples). The samples were grouped by the eight breeds (K = 8). In the case of K = 7, YT and BYT breeds were grouped in one cluster.

We used 21 STRs to determine the genetic population structure of eight canine breeds chosen for this study. Relationships of breeds were analyzed by two approaches—the Reynolds genetic distance, which provides the highest sensitivity for highly divergent populations [21,30], and model-based clustering [19]. The results of STRUCTURE confirmed that the eight dog breeds analyzed could be subdivided into eight genetic clusters. The results for K = 7, for Biewer Yorkshire Terrier and Yorkshire Terrier breeds, showed one cluster (Figure 4).

The two clusters formed by BYT and YT are suggestive of genetic sub-structuring that resulted from using animals belonging to divergent selection lines. The Biewer breed was founded in Germany in 1984 as a result of the selection for white hair genes to produce tricolor dogs. The Biewer Yorkshire Terriers were ultimately recognized as a distinct breed in 1989 by the Allgemeiner Club der Hundefreunde Deutschland (ACH). The Biewer Terrier became the first breed proven to be distinct and unique using genetic testing [31]. This breed became recognized in 2021 by the AKC as the organization’s 197th breed [5]. The estimated genetic distance confirmed the genetic similarity between YT and BYT (θ_w_ = 0.26). Both breeds showed genetic closeness to Maltese (θ_w_ < 0.4), which is considered, alongside the Black and Tan Terrier and Skye Terrier breeds, as the ancestor of the Yorkshire Terrier. Similar genetic proximity (θ_w_ = 0.41) was observed between Maltese and French Bulldog. The genetic distance between the other breeds was θ_w_ > 0.50. The differences between GR and LR, which belong to the same group of retrievers and flushing dogs (pointer type), may be due to their different origins. Golden Retriever is a breed developed in the 19th century in Great Britain, while Labrador Retriever dates back from the 18th century and comes from Newfoundland. Labrador Retriever shows similar genetic distance (θ_w_ = 0.55) with German Shepherd. Phylogenetically the most distant breed is Irish Wolfhound which originated in Ireland and was used to hunt and protect against wolves. The breed became extinct as wolf numbers decreased and post-19th century it was presumably recreated by dog fanciers [32].

The highest within-breed diversity was characteristic of the M, BYT, and YT breeds, in which the largest number of alleles was identified. In Maltese, 4 out of 135 identified alleles were specific for this breed, but their frequency was low (less than 3%). In contrast, a 128 bp allele at the CXX279 locus, common only to BYT and YT, was present in both breeds with a frequency of more than 16%. For these breeds, the highest degree of heterozygosity (over 65%) was also noted. For Yorkshire Terrier, Ho = 0.662, which is similar to the studies in the UK [8] and the US [33], where Ho for this breed was 0.66 and 0.789, respectively, indicating good levels of diversity in the YT breed. For the other breeds (GS, GR, LR, and FB), the degree of heterozygosity exceeded 50%. The genetic analysis of GS, which was performed based on the same STR panel and using a different 15-STR panel, showed similar values as in our study: Ho = 0.56 and Ho = 0.54, for dogs from the UK and Italian, respectively [8,9]. Tahir et al. [34], who used a 15-STR panel for GS breed in Pakistan, showed H_O_ to be 0.742. In the same study, higher values were also observed for the Labrador Retriever breed (H_O_ = 0.675) than in our study. A degree of heterozygosity below 50% was observed only for IW. This breed was characterized by the lowest total number of identified alleles. Furthermore, only two alleles each were determined in as many as four loci, among which 131 bp (AHTh130) and 216 bp alleles (REN169D01) had a frequency of 0.912 and 0.945, respectively. The mean effective number of alleles per locus was only 2.1. Limited genetic variation may be the result of the origin of this breed from a common founder population involving four founder lines [35]. Preliminary studies for Irish Wolfhound reported by UC Davis Veterinary Genetics Laboratory showed that H_O_ = 0.502 for 33 STR, and when a 21-STR panel was used, H_O_ = 0.483, and this value was similar to that obtained in our study (H_O_ = 0.491) [36]. In the breeds under study, the mean H_O_ and H_E_ values were similar to one another. The inbreeding coefficient for five breeds was negative, but the mean F_IS_ had low negative values (from −0.004 to −0.049), which suggests no inbreeding in these breeds. The lack of observed genetic bottlenecking in any breed, despite a breeding system, may be due to multiple pedigree lines used. The lack of inbreeding was also reported in Bracco Italiano (F_IS_ = 0.061), Tatra Shepherd (F_IS_ = −0.005), six livestock guard dog breeds (average F_IS_ value = 0.024), and in Polish Hunting Dog (F_IS_ = −0.01) [9,11,13,36].

The usefulness of the studied markers for individual identification of dogs was evaluated based on standard PIC, PD, and P_ID_ genetic parameters. As reported by Botstein et al. [25], markers with PIC values greater than 0.5 are considered to be informative, values between 0.25 and 0.50 are fairly informative, and values lower than 0.25 are not very informative. In our study, the mean PIC values were greater than 0.5 for most of the studied breeds, similar to many other breeds of dogs investigated [11,13,34,37,38,39,40]. Only in the GS and IW breeds were slightly lower PIC values (0.49 and 0.41, respectively) obtained (Table 4). An immediate measure of the usefulness of the analyzed STR panels for individual identification is the power of discrimination. The higher the power of discrimination of a given STR panel is, the greater the chance that it can be used for individual identification [41]. The PD for the panel of 16 microsatellite markers for Shiba Inu breed was more than 0.999999 [8], while cumulative PD for the 21 markers in all breeds in our study was close to 1.0. The 21-STR panel used for the human gave a similar value of 0.999999999999999999999999999967 [42]. The probability of identity was calculated to assess the suitability of tested panels for individual identification. P_ID_ shows the probability with which two unrelated, randomly selected individuals in the population will have the same genotype. It is accepted that CP_ID_ values ranging between 10^−3^ and 10^−4^ are sufficiently low for the identification of individuals in natural animal populations [43], whereas CP_ID_ value estimated only for 15 STR markers in canine amounted to 10^−8^ [44]. In our study, CP_ID_ ranged between 10^−11^ for IW and 10^−19^ for M and YT. For all breeds, the obtained low CP_ID_ values should be sufficient to distinguish individual dogs. The usefulness of the investigated panel of markers for parentage verification was determined by calculating the probability of exclusion (PE). For a previously used commercial panel of 10 STR, CPE was 0.994 [45]. The use of 17 or 18 STR markers gave a CPE of 0.99998%, and 0.99996%, respectively [11,46]. For all breeds, the recommended panel of 21 STR, used in this study, achieved a CPE_1_ above 0.99 and CPE_2_ above 0.999, except for GS (CPE_1_ = 0.986) and IW breeds (CPE_1_ = 0.949, CPE_2_ = 0.998). The values higher than CPE_1_ of 0.9999 and CPE_2_ of 0.99999 were obtained for YT, M, and BYT. The highest CPE_2_ = 0.999999 was observed for YT.

## 5. Conclusions

Analysis of the microsatellite DNA markers provides valuable information about canine diversity, and a 21-STR panel is an effective tool for individual identification and parentage testing. Our study showed the lowest PIC (0.414), PE_1_ (0.949), and PE_2_ (0.998) with the highest P_ID_ (6.71 × 10^−11^) for the Irish Wolfhound breed, illustrating the lower effectiveness of the STRs panel. In contrast, Yorkshire Terrier and Maltese breeds had the highest PIC (0.655 and 0.640), PE_1_ (0.9995 and 0.9994), and PE_2_ (0.999999 and 0.9999987) with the lowest P_ID_ (2.34 × 10^−19^ and 4.47 × 10^−19^). The results suggest the popular breeds in Poland have sufficient diversity relative to other populations that have been studied. The research here provides baseline data for monitoring and managing the breeds.

## Figures and Tables

**Table 1:** Reynolds genetic distance (θ_w_) values of eight study dog breeds.

Breed	FB	GR	LR	M	GS	IW	YT	BYT
BF	0.000							
GR	0.540	0.000						
LR	0.469	0.577	0.000					
M	0.409	0.501	0.459	0.000				
GS	0.519	0.596	0.549	0.459	0.000			
IW	0.574	0.606	0.588	0.539	0.634	0.000		
YT	0.430	0.503	0.465	0.364	0.484	0.500	0.000	
BYT	0.435	0.511	0.485	0.372	0.511	0.510	0.260	0.0000

**Table 2:** The number of alleles identified per locus (N), mean number of alleles per locus (A), and mean effective number of alleles per locus (Ae) for each breed.

Locus	GS		M		IW		BYT		YT		GR		LR		FB		N
	A	Ae	A	Ae	A	Ae	A	Ae	A	Ae	A	Ae	A	Ae	A	Ae	
AHT121	7	1.8	9	4.9	5	3.1	9	4.5	8	5.2	6	2.4	7	2.4	8	4.6	11
AHT137	7	2.0	6	4.5	2	1.2	9	4.1	7	3.4	5	4.5	9	3.2	7	4.0	12
AHTh171	7	2.7	9	2.5	5	2.4	5	1.6	9	4.7	3	2.7	7	3.8	4	2.4	11
AHTh260	8	2.6	6	4.0	3	1.6	8	3.5	6	3.7	4	1.4	9	3.1	6	2.5	10
AHTk211	4	2.8	5	3.4	3	1.9	6	2.9	5	1.6	4	2.0	6	2.1	4	2.0	6
AHTk253	6	1.5	5	2.5	3	1.9	6	2.8	5	3.3	3	1.8	5	2.0	4	3.2	7
CXX279	5	2.8	4	3.4	5	2.2	8	3.5	8	3.5	4	1.3	8	3.1	5	2.6	8
FH2054	6	3.3	8	5.3	5	2.4	6	3.9	6	3.2	6	3.4	7	2.7	8	4.0	8
FH2848	6	2.2	6	2.2	3	2.2	7	3.1	5	3.3	3	1.8	4	3.9	5	2.8	8
INRA21	6	3.0	5	3.8	4	2.9	6	3.8	6	3.5	4	2.6	5	3.1	5	1.4	8
INU005	4	2.3	5	1.5	3	2.4	6	1.3	8	3.2	5	3.5	5	2.0	5	2.8	9
INU030	5	2.1	6	2.6	2	1.7	6	2.5	7	3.2	3	2.7	4	1.8	3	1.8	7
INU055	5	3.0	5	3.8	4	1.7	6	2.6	6	2.6	4	2.3	5	1.9	2	2.0	7
REN162C04	5	2.4	6	1.9	3	2.1	7	3.4	7	3.4	4	1.6	5	2.1	3	1.7	8
REN169D01	3	1.9	8	3.7	2	1.1	5	2.9	6	3.1	4	3.6	7	3.3	4	3.0	8
REN169O18	10	4.3	5	2.4	4	2.1	6	2.4	5	3.2	6	3.3	5	2.3	5	3.1	12
REN247M23	4	1.5	5	3.3	3	2.2	4	3.4	4	3.4	5	1.6	3	1.3	4	2.7	7
REN54P11	5	2.1	8	5.4	3	1.9	7	4.4	6	3.8	7	3.7	6	2.9	6	3.0	9
AHTh130	7	3.8	10	5.1	2	1.3	8	3.5	5	3.0	3	2.6	6	3.9	8	3.6	10
REN105L03	8	2.3	7	4.7	4	2.6	7	3.9	5	4.0	5	4.0	4	1.5	5	3.0	10
REN64E19	7	1.2	7	2.2	4	2.8	6	3.2	7	3.7	4	2.7	5	3.2	5	4.2	9
N	125		135		72		138		131		92		122		106		185
A	5.9		6.4		3.4		6.6		6.2		4.4		5.8		5.1		
Ae	3.3		3.5		2.1		3.2		3.5		2.6		2.6		2.9		

**Table 3:** Frequency of private alleles in the study breeds.

Locus	Allele (bp)	GS	M	BYT	YT	GR	LR	FB
ATH121	92		0.031					
ATH137	127				0.0130			
AHTH260	256						0.0243	
AHTK253	296	0.0354						
CXX279	128			0.1641 *	0.1753 *			
FH2848	234					0.0208		
INRA21	109		0.0185					
INU005	134				0.0454			
REN162C04	212	0.1982						
REN169O18	156							0.1197
	158	0.2168						
	176	0.0097						
	178	0.0044						
REN247M23	274							0.0128
	276						0.0146	
REN54P11	240		0.0062					
AHTH130	117		0.0123					
REN105L03	229							0.2179
	245	0.0079						
REN64E19	159			0.0115				

* Alleles common to the Biewer Yorkshire Terrier (BYT) and Yorkshire Terrier (YT) breeds only.

**Table 4:** Mean values of genetic parameters assessed for 21 STR loci of the study breeds.

Breed	H_O_	H_E_	F_IS_	*p*-Value	PIC	CPD	CP_ID_	CPE_1_	CPE_2_
GS	0.5451	0.5541	0.0171	0.4840	0.4941	1 *	1.80 × 10^−13^	0.985991	0.9997326
M	0.6855	0.6771	−0.0127	0.4907	0.6398	1 *	4.47 × 10^−19^	0.999443	0.9999987
IW	0.4911	0.4743	−0.0373	0.3952	0.4139	1 *	6.71 × 10^−11^	0.949242	0.997768
BYT	0.6608	0.6581	−0.0041	0.3425	0.6166	1 *	6.82 × 10^−18^	0.99875	0.999996
YT	0.6623	0.6981	0.0533	0.3150	0.6545	1 *	2.34 × 10^−19^	0.999495	0.999999
GR	0.5922	0.5620	−0.0490	0.4414	0.5135	1 *	3.37 × 10^−14^	0.99010	0.999875
LR	0.5954	0.5886	−0.0088	0.2813	0.5429	1 *	4.38 × 10^−15^	0.99316	0.999941
FB	0.6077	0.6177	0.0173	0.5395	0.5602	1 *	8.65 × 10^−16^	0.99601	0.999964

* actual value < 1.0, equal to approximately > 0.9999999.

## Data Availability

The data presented in this study are available within the article.
